# Future of spine research: “The Asian perspectives”

**DOI:** 10.1002/jsp2.1019

**Published:** 2018-06-27

**Authors:** Daisuke Sakai, Robert Mauck, Mauro Alini

**Affiliations:** ^1^ Department of Orthopaedic Surgery Tokai University School of Medicine Isehara Japan; ^2^ McKay Orthopaedic Research Laboratory University of Pennsylvania School of Medicine Philadelphia Pennsylvania; ^3^ AO Research Institute Davos Switzerland

The Editors are delighted to deliver the second issue of JOR Spine. Great appreciation to authors and reviewers for their dedication and service.

Like any other field in medicine, we face great change in demographics in the origin of submitted articles in spine research, with the vast numbers coming from Asia. Today, China is second only to the United States in terms of English scientific publication output, and by 2019, it is estimated to take over the lead from United States, Europe, and Japan. There is no doubt that scientific community will also see more research manuscripts coming from India and Southeast Asia as well. There is still prominent difference in ways of thinking within the new members and such difference leading to large number of misconduct, fabrication, and so on^1^; however, we must admit that in parallel, the amount of high‐impact research with marked contribution in advancement of science is also rapidly growing.

One of the trend to note in scientific publication coming from China is a 200‐fold increase from 2001 to 2011 in meta‐analysis and systematic reviews in various topics^2^. It is difficult to understand the reason for many researchers demanding to perform a systematic review than original articles, but this can be concluded that it is most likely due to the culture in Asian academia, which becomes uncontrollably competitive, and the number of papers published is the most important benchmark for scientists' and clinicians' promotion. It can be forecasted that this trend will continue until next significant change in the society system occurs in Asian academia.

To date, significant work in spine research has been generated from Asia. For example, research associated with spinal pain has been extensively reported from Asia. Asia has led the field in intervertebral disc biology, especially in stem cell research for the past decade. Regenerative therapies have become the hot topic and it is sure that research and development of regenerative medicinal products with the use of iPS technology shall also gain attention. Furthermore, translational and clinical studies shall also accelerate in Asia, with the new Japanese law effective November 2014, surpassing the clinical development process of regenerative medicine, by treating regenerative medicine products in a similar manner to orphan drugs. This means that the approved product will typically skip the Phase III trial and obtain marketing authorization after demonstration of safety and minimal signs of efficacy after a solid Phase I and II trial. This fast‐track system is not available anywhere else and has gained attention from various authorities around the globe. It goes without saying that we can keep an eye on Asia.
